# Adjuvant therapy with Huatan Sanjie Granules improves the prognosis of patients with primary liver cancer: a cohort study and the investigation of its mechanism of action based on network pharmacology

**DOI:** 10.3389/fphar.2023.1091177

**Published:** 2023-05-30

**Authors:** Juhua Yuan, Abdusami Abdurahman, Ning Cui, Tengteng Hao, Jianhua Zou, Liren Liu, Yu Wu

**Affiliations:** ^1^ Beijing Hospital of Integrated Traditional Chinese and Western Medicine, Beijing, China; ^2^ Key Laboratory of Cancer Prevention and Therapy, National Clinical Research Center for Cancer, Tianjin’s Clinical Research Center for Cancer, Department of Molecular Pharmacology, Tianjin Medical University Cancer Institute and Hospital, Tianjin, China; ^3^ Xiyuan Hospital, China Academy of Chinese Medical Sciences, Beijing, China

**Keywords:** Huatan Sanjie Granules (HSG), primary liver carcinoma (PLC), network pharmacology, PI3K-Akt/MAPK signaling pathways, adjuvant therapy

## Abstract

**Objective:** Nowadays, primary liver carcinoma (PLC) is one of the major contributors to the global cancer burden, and China has the highest morbidity and mortality rates in the world. As a well-known Chinese herbal medicine (CHM) prescription, Huatan Sanjie Granules (HSG) has been used clinically for many years to treat PLC with remarkable efficacy, but the underlying mechanism of action remains unclear.

**Methods:** A clinical cohort study was conducted to observe the overall survival of PLC patients with vs. without oral administration of HSG. Meanwhile, the BATMAN-TCM database was used to retrieve the potential active ingredients in the six herbs of HSG and their corresponding drug targets. PLC–related targets were then screened through the Gene Expression Omnibus (GEO) database. The protein–protein interaction (PPI) network of targets of HSG against PLC was constructed using Cytoscape software. The cell function assays were further carried out for verification.

**Results:** The results of the cohort study showed that the median survival time of PLC patients exposed to HSG was 269 days, which was 23 days longer than that of the control group (HR, 0.62; 95% CI, 0.38–0.99; *p* = 0.047). In particular, the median survival time of Barcelona Clinic Liver Cancer stage C patients was 411 days in the exposure group, which was 137 days longer than that in the control group (HR, 0.59; 95% CI, 0.35–0.96; *p* = 0.036). Meanwhile, the enrichment analysis result for the obtained PPI network consisting of 362 potential core therapeutic targets suggest that HSG may inhibit the growth of liver cancer (LC) cells by blocking the PI3K-Akt/MAPK signaling pathways. Furthermore, the above prediction results were verified by a series of *in vitro* assays. Specifically, we found that the expressions TP53 and YWHA2, the targets of the hepatitis B virus signaling pathway, were significantly affected by HSG.

**Conclusion:** HSG shows promising therapeutic efficacy in the adjuvant treatment of PLC.

## 1 Introduction

Primary liver carcinoma (PLC) is a common malignant tumour of the digestive system worldwide (Gao et al., 2020).According to the new data released by GLOBOCAN 2018, the annual number of new cases of PLC in the world ranks 6^th^ among malignant tumours, and the number of deaths ranks 2^nd^, the morbidity and mortality rates in China reached 55.4% and 53.9%, respectively (Ferlay et al., 2019). Overall, the prognosis of PLC patients is extremely poor, and the ratio of morbidity to mortality is 1:0.9. Surgical treatment (including surgical resection and liver transplantation) is still the preferred treatment method for early-stage PLC patients and the only treatment that can enable patients to achieve long-term survival. As a systemic treatment, traditional Chinese medicine (TCM) is used throughout the whole course of PLC, especially as a palliative treatment for advanced PLC (Bruix et al., 2016; Ayuso et al., 2018; Marrero et al., 2018; Benson et al., 2021).

In TCM system, it is believed that the formation of PLC is mainly caused by “sputum poisoning” (Yuan et al., 2017). The principles of medication under TCM usually include treatments such as clearing heat and resolving phlegm, drying dampness and resolving phlegm, and activating QI and resolving phlegm. TCM prescriptions have received increasing attention due to such advantages as fewer toxicity and side effects and good drug safety (Li et al., 2019; Zeng et al., 2019) HSG is a classic prescription for the treatment of PLC proposed by Xiyuan Hospital of China Academy of Chinese Medical Sciences based on the aetiology of “sputum poisoning” and decades of clinical experience of renowned TCM doctors. HSG is composed of six CHMs, including *Pleiones Pseudobulbus* (Chinese name: Shan Ci Gu, SCG), *Arisaema Cum Bile* (Chinese name: Dan Nan Xing, DNX), *Curcumae Longae Rhizoma* (Chinese name: Jiang Huang, JH), *Curcumae Radix* (Chinese name: Yu Jin, YJ), *Bolbostemmatis Rhizoma* (Chinese name: Tu Bei Mu, TBM), and *Fritillariae Thunbergii Bulbus* (Chinese name: Zhe Bei Mu, ZBM). These six CHMs have strong capabilities of phlegm resolution, detoxification, and resolving masses. Among them, SCG has the functions of clearing heat and detoxication, phlegm resolution, and resolving masses. JH, YJ, and DNX all have the capabilities of phlegm resolution and resolving masses, among which JH and YJ tend to activate *qi* and resolve phlegm, DNX tends to dry dampness and resolve phlegm. ZBM and TBM both have the capabilities of clearing heat, resolving phlegm, and resolving masses.

In recent years, phlegm-resolving CHMs have gradually received attention and been applied in the clinical treatment of PLC. Some of the above six phlegm-resolving herbs and their active ingredients or extracts have now been shown to have inhibitory effects on LC cells (Darvesh et al., 2012; XU et al., 2015; Chao et al., 2019). However, TCM treatment is a systemic process, and there are synergistic or antagonistic effects between various ingredients or their targets. Traditional pharmacological methods have obvious defects in the study of TCM prescriptions. With the advancement of bioinformatics, network pharmacology has recently become a useful tool for studying the mechanisms-of-action of complex systems of TCM.

In this research, we started with a cohort clinical study and confirmed that HSG had a good therapeutic effect on patients with PLC. Next, a virtual study was conducted through the combination of TCM network pharmacology and bioinformatic methods to explore the underlying mechanism-of-action of HSG in the treatment of PLC. Most importantly, we further verified the effect of HSG on the biological functions and signaling pathways of PLC cells through biological experiments. Therefore, our research combined network pharmacology with biological functional assays may provide a new idea for exploration of clinical uses of classic TCM prescriptions (Zhang et al., 2022).

## 2 Materials and methods

### 2.1 To participate in experimental patient conditions and intervention programs

The patients in this study were from Xiyuan Hospital, China Academy of Chinese Medical Sciences and No. 309 Hospital of Chinses People’s Liberation Army and had been diagnosed with primary liver cancer. Inclusion criteria: 1) age ≥18 years; 2) an estimated survival of more than 2 months; 3) the Eastern Cooperative Oncology Group(ECOG) score≥4; 4) The decoction can be administered orally or through a stomach tube. Exclusion criteria: 1) severe liver cancer complications within the past month, such as upper gastrointestinal bleeding, hepatic encephalopathy, liver cancer nodule rupture and bleeding or uncontrolled infection; 2) severe cardiovascular disease, severe kidney or brain diseases; 3) current breastfeeding or pregnancy. All patients signed informed consent and all study procedures were approved by the Ethics Committees of the Xiyuan Hospital, China Academy of Chinese Medical Sciences.

Treatment plans: The exposure group received TCM treatment with HSG as the main prescription on top of conventional Western medicine treatment. The TCM treatment was given for at least 2 months. Patients in the nonexposure group were treated with conventional Western medicine treatment only. Both the exposure group and the nonexposure group were received the same basic Western medicine treatment which includes the surgery, radiotherapy, chemotherapy, targeted therapy, or other local treatments such as interventional therapy and radiofrequency therapy according to clinical needs followed up at least once a month by outpatient visits, telephone, or mail, until the end of the study. The evaluation of the stage summary included the survival status, efficacy analysis, safety evaluation, and ECOG score. The study endpoint was based on overall survival. Death was defined as liver cancer–related death. The follow-up period was 30 months from 1 July 2012 to 1 January 2015.

### 2.2 Predictive analysis of traditional Chinese medicine network pharmacology combined with bioinformatics

#### 2.2.1 Screening of potential active ingredients of HSG

According to the BATMAN-TCM online database (http://bionet.ncpsb.org.cn/batman-tcm/), the potential active ingredients of six CHMs contained in HSG (SCG, JH, ZBM, YJ, DNX, and TBM) were obtained. The screening parameters were all the system default values (i.e., score cut-off: 20; adjusted *p*-value: 0.05).

#### 2.2.2 Prediction of the targets of the potential active ingredients of Huatan Sanjie Granules

Next, the corresponding drug targets of the potential active ingredients of HSG were obtained by searching the BATMAN-TCM database. The UniProt online database (https://www.uniprot.org/) was queried to verify each target to confirm that the source genus was *Homo*. Then, we used this information to fill out a drug target dataset.

#### 2.2.3 Screening the disease targets associated with primary liver carcinoma

The retrieved PLC-related targets were identified by searching in the Gene Expression Omnibus database (https://www.ncbi.nlm.nih.gov/geo/). The screening criteria were as follows: fold change (FC): 2; adjusted *p*-value: 0.05. Differentially expressed genes were analyzed using GEO2R. The data obtained in this manner included three gene expression microarray datasets (GSE84402, GSE45267, and GSE101685) from human PLC as well as paracancerous tissues.

#### 2.2.4 Systematic network construction and enrichment analyses

Through the BisoGenet plugin of Cytoscape (version 3.2.1) software, a protein–protein interaction (PPI) network between HSG-related drug targets and PLC–related disease targets were constructed, and the networks were then visualized. The above two PPI networks were further merged, and the topological parameters of each node in this merged network were calculated using CytoNCA, another plugin of Cytoscape software. The key nodes in this merged network were screened, which were those nodes with higher than two times the median degree centrality (DC), and a new network was generated from these nodes. Next, to further screen the potential core targets of HSG against PLC, the nodes with topological parameters higher than the median DC, betweenness centrality (BC), and closeness centrality (CC) in the new network were mined (Pan et al., 2018). And these targets were considered to be the most significant among all nodes. Enrichment analyses of the biological functions and signaling pathways of the obtained potential core targets were performed using the Database for Annotation, Visualization and Integrated Discovery (DAVID) v6.8 (https://david.ncifcrf.gov/) online database, respectively.

### 2.3 Biological experimental verification

#### 2.3.1 Cell cultures and related reagents

The human LC cell HepG2, Hep3B, and Huh-7 cell lines were purchased from China Infrastructure of Cell Line Resources (School of Basic Medicine Peking Union Medical College, China) and cultured in Dulbecco’s modified Eagle medium containing 10% (v/v) FBS and 100 U/ml streptomycin/penicillin at 37°C and 5% CO_2_. Cell Counting Kit-8 (CCK-8) reagent was purchased from Dojindo (Japan). Hoechst 33,342 reagent was purchased from Beyotime (China). An Annexin-V FITC apoptosis detection kit (#556547) and BrdU cycle detection kit Part A (#559619) were purchased from BD Biosciences Pharmingen (United States). Antibodies against Bcl-2, Bax, Caspase 9/p35/p10, cyclin D1, and beta-actin were purchased from Proteintech (United States). Antibodies against cyclin A2, cyclin B1, p-Akt(S473), p-Akt(T308), pan-AKT, p-ERK (T202/Y204), and pan-ERK were purchased from Cell Signaling Technology (United States). Anti-CDK2 antibody was purchased from Abcam (United Kingdom). HSG was provided by Beijing Kangrentang Pharmaceutical Co., Ltd. The concentration of the prepared HSG mother solution was 0.1 g/mL. For the biological experiments, the 0.1 g/mL stock decoction could be subjected to 0.22-µm filter-sterilization and dilution as needed.

#### 2.3.2 Cell pharmacodynamics and biological function validation

Cell pharmacodynamic evaluation was performed using the CCK-8 assay, scratch assay, and plate cloning assay to examine the cell growth ability. Especially, in cell colony formation assay, we first treated HepG2 and Huh-7 cells with 0.5 times IC_50_ and IC_50_ of HSG for 24 h, respectively. Then, we used the complete culture medium without drugs, and the cells (HSG treatment groups and blank control group) were further carried out for 14 days and cell colonies were counted after being stained with 0.1% crystal violet. Cell apoptosis and cell cycle detection were used to verify the biological functions. The specific experimental method has been described in detail previously (Pan et al., 2018).

#### 2.3.3 Western blot analysis

Cells in each experimental group in the logarithmic growth phase were collected, and an appropriate amount of RIPA lysis buffer was added. The cells were lysed on ice for 10 minues and then centrifuged at 13,000 × *g* for 10 min at 4°C. The supernatant was collected, and the precipitate was discarded. The protein concentration was determined by the standard bicinchoninic acid method. Each protein sample was mixed with 5× sodium dodecyl sulfate gel loading buffer. After denaturing the protein at 100°C for 5 min, the samples were subjected to 10% SDS-polyacrylamide gel electrophoresis, then transferred to polyvinylidine fluoride (PVDF) membranes at 90 V for 100 min. Nonspecific antibodies in the membrane were blocked with TBS-T containing 5% skim milk powder for 1 h at room temperature. The PVDF membrane was washed three times, and the corresponding primary antibody was added to the membrane in buffer and incubated at 4°C overnight. The PVDF membrane was washed again three times the next day. The corresponding secondary antibody was added and incubated at room temperature for 1 h. A gel imaging system was used for scanning. Meanwhile, the statistical analysis on the grayscale values of the corresponding bands by using “ImageJ” software.

#### 2.3.4 Real-time quantitative reverse transcription–PCR (qRT-PCR) assay

The target cell RNA was extracted according to a kit manual and reverse-transcribed into cDNA. All reaction mixes were 25 μL. The program settings were as follows: 95°C for 3 min; 35 cycles of 95°C for 20 s and 56°C for 20 s; then 72°C for 20 s, 95°C for 15 s, 60°C for 15 s, 95°C for 20 min, and 95°C for 15 s. The specific primer information is in Table 1.

**TABLE 1:** qRT-PCR primer information.

Gene name	The primer
TP53	F:CAGCACATGACGGAGGTTGT
	R:TCATCCAAATACTCCACACGC
GRB2	F:CTGGGTGGTGAAGTTCAATTCT
	R:GTTCTATGTCCCGCAGGAATATC
CDK2	F:CCAGGAGTTACTTCTATGCCTGA
	R:TTCATCCAGGGGAGGTACAAC
YWHAZ	F:CCTGCATGAAGTCTGTAACTGAG
	R:GACCTACGGGCTCCTACAACA
MYC	F:GGCTCCTGGCAAAAGGTCA
	R:CTGCGTAGTTGTGCTGATGT

### 2.4 Statistical analysis

Data were statistically processed with GraphPad Prism v. 6.0.1 (GraphPad Software, San Diego, CA, United States). Measurement data aredescribed as the mean ± standard deviation (±s). Data conforming to a normal distribution were analysed by one-way analysis of variance. Data with a nonnormal distribution were analysed by the rank sum test. Count data are described as the rate or composition ratio, and groups were compared by the chi-squared test. Kaplan-Meier method was used for survival analysis, and log-rank test, Cox regression model were used to analyze the related factors. *p* < 0.05 was considered a statistically significant difference.

## 3 Results

### 3.1 General data of patients participating in the clinical study

The study started from July 2012 to the end of follow-up in June 2015, a total of 102 patients were enrolled in strict accordance with the inclusion criteria, but five were lost to follow-up. A total of 97 patients were included in the analysis. There were a total of 50 patients (two were lost to follow-up) in the exposure group, and they were from the Xiyuan Hospital, China Academy of Chinese Medical Sciences. There were 47 patients in the nonexposure group (three were lost to follow-up), and they were from No. 309 Hospital of Chinses People’s Liberation Army. The general clinical data of the two groups of patients are shown in (Table 1). There were no significant differences between the two groups in terms of sex, age, type of PLC diagnosis, history of hepatitis, presence of portal vein tumour thrombus, BCLC(Barcelona Clinic Liver Cancer), presence of distant metastasis, ECOG score, or Child‒Pugh classification (*p* > 0.05). Thus, the baseline data of the two groups were consistent (Table 2).

**TABLE 2:** General data of exposure and nonexposure group (cases).

Category	Characteristics	Exposure group	Nonexposure group
Gender	Male	37	40
	Female	13	7
Median Age/Year	∼	58 ± 11	55 ± 11
Diagnosis of type	Hepatocellular carcinoma	29	32
	Cholangiocarcinoma	6	3
	Clinical diagnosis	15	12
Portal vein carcinoma thrombus	Yes	24	26
	No	16	31
Distant metastases	Yes	15	17
	No	35	30
History of viral hepatitis	Yes	37	13
	No	40	7

### 3.2 Survival analysis

#### 3.2.1 Overall survival

**FIGURE 1:**
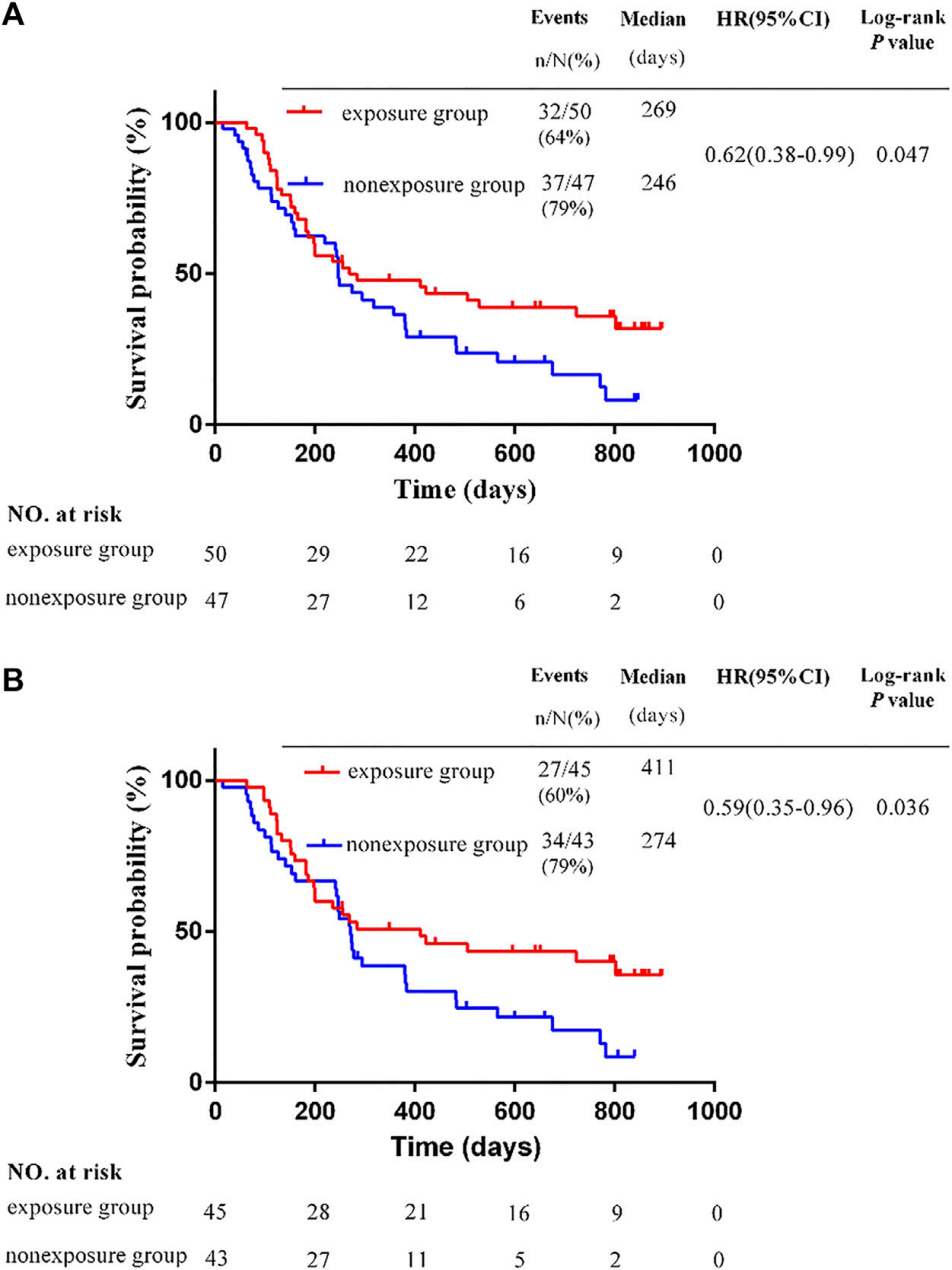
Kaplan-Meier survival estimates for progression-free survival. **(A)** Overall survival in the whole study. **(B)** Overall survival of patients with different BCLC stage C *p < 0.05 by the Student's *t*-test.

The OS of the exposure group was was significantly longer than that of the nonexposure group. The mOS was 269 days in the exposure group, and 246 days in the nonexposure group (HR, 0.62; 95% CI, 0.38–0.99; *p* = 0.047) (Figure 1A).

#### 3.2.2 Overall survival of patients with different barcelona clinic liver cancer stages

In this study, all patients were BCLC stage C or D, and no patients were stage A or B. Among patients with stage C, there were 45 patients in the exposure group and 43 in the non-exposure group. The mOS in the exposure group was 411 days, and 274 days in the non-exposure group. The mOS in the exposure group was significantly longer than that of the nonexposure group (HR, 0.59; 95% CI, 0.35–0.96; *p* = 0.036) (Figure 1B). Among patients with stage D, there were 5 patients in the exposure group and 4 in the non-exposure group. Due to the insufficient sample size in the group of patients with stage D, it is not meaningful to conduct statistical analysis. The survival time of 9 cases in two groups of patients with stage D are shown in Table 3.

**TABLE 3:** Survival time of 9 cases in two groups of patients with stage D.

Group	Case 1	Case 2	Case 3	Case 4	Case 5	Case 6	Case 7*	Case 8	Case 9
exposure	106	94	82	165	529				
nonexposure						157	64	61	67

*lost to follow-up.

#### 3.2.3 Security

No serious adverse events occurred in either group during the trial. The incidence of treatment-related adverse events (TRAEs) including leukopenia, nausea, diarrhea, fatigue, and appetite loss in the exposure group were lower than that in nonexposure group, which is shown in Table 4.

**TABLE 4:** Summary of TRAEs.

Treatment-related adverse events	Exposure group (n = 50)	Nonexposure group (n = 47)	*p*-Value
Leukopenia	10 (20%)	19 (40%)	0.045
Hepatotoxicity	16 (32%)	14 (30%)	0.830
Nausea	20 (40%)	29 (62%)	0.042
Diarrhea	9 (18%)	20 (43%)	0.014
Fatigue	16 (32%)	27 (57%)	0.015
Appetite loss	18 (36%)	28 (60%)	0.026
Hair loss	7 (14%)	12 (26%)	0.202
hand-foot syndrome	13 (26%)	17 (36%)	0.380
Rash	12 (24%)	10 (21%)	0.811
Hypertension	11 (22%)	14 (30%)	0.487

### 3.3 The candidate active ingredients and potential drug targets of Huatan Sanjie Granules

**FIGURE 2:**
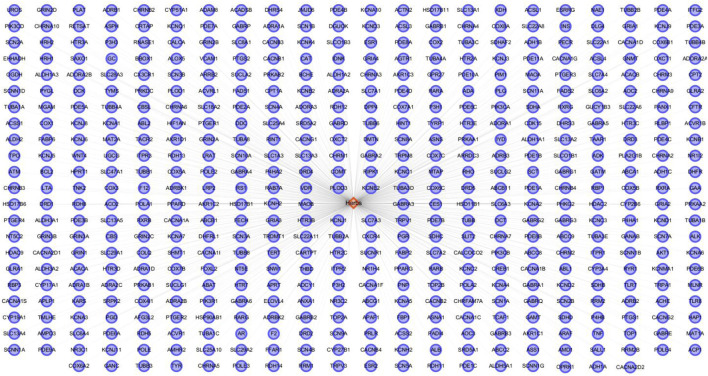
Construction of the HSG ingredients and drug targets network. The network was established by connecting the candidate key ingredients in *HSG* and their putative targets.

Using the BATMAN-TCM database, the relevant potential active ingredients in HSG were searched, and a total of 22 ingredients were obtained. Its targets were still screened through the BATMAN-TCM database. A total of 500 protein targets were obtained by pooling and de-duplicating. The results showed that there were many overlapping targets between HSG’s different ingredients, indicating that these compounds may play key roles in the synergistic effects. Then, cytoscape software was used to construct a drug-target network to visualize the interactions between the systems (Figure 2).

### 3.4 Construction of the protein–protein interaction network and enrichment analyses of the core targets of Huatan Sanjie Granules against primary liver carcinoma

**FIGURE 3:**
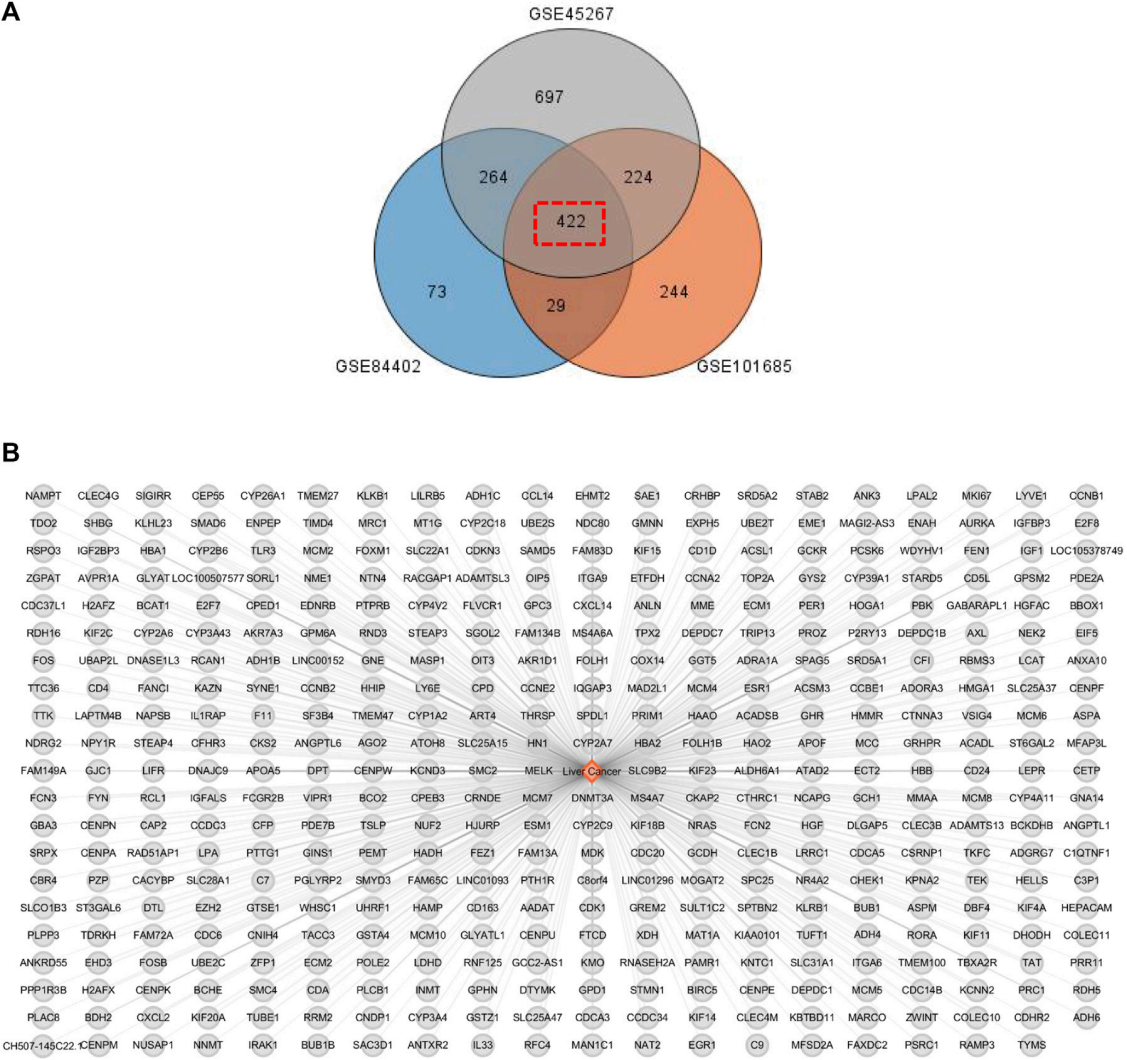
PLC-related disease targets were collected from the GEO database. **(A)** The relevant data from GEO chips, including GSE84402, GSE45267, and GSE101685. Venn diagram composed of 422 overlapped PLC-related targets from above three GEO chips. **(B)** PLC-associated disease targets network construction.

**FIGURE 4:**
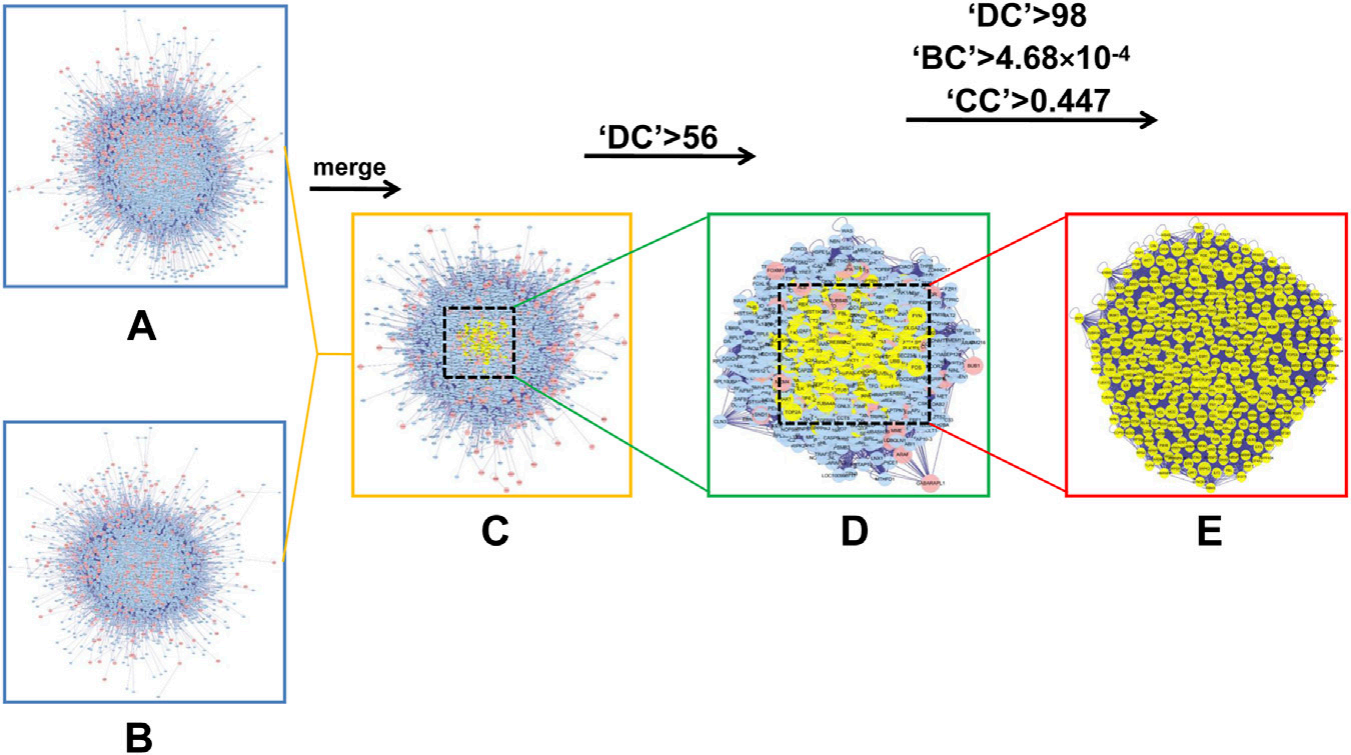
In silico evaluation and network construction of the core targets for HSG treatment of PLC. **(A)** Drug target PPI network of *HSG* was composed of 7,121 nodes and 152,906 edges. **(B)** PPI network of PLC-associated disease targets was made of 6,126 nodes and 134,394 edges. **(C)** Integrative PPI network of *HSG* in PLC was made up of 4,098 nodes and 102,784 edges. **(D)** PPI network of the key targets extracted from c, in which 1,071 nodes and 46,746 edges was obtained. **(E)** PPI network containing the core targets extracted from d, including 362 nodes and 13,645 edges were shown.

Since previous clinical studies have shown that HSG has a good therapeutic effect on PLC, we began to explore its underlying mechanisms for the treatment of PLC. We further used the BisoGenet plugin of cytoscape software to perform PPI analysis on the 500 previously obtained potential drug targets by using network pharmacology and bioinformatic techniques. The network had 7121 nodes and 152,906 edges. The gene expression results of three microarrays (GSE84402, GSE45267, and GSE101685) of human-derived PLC and paired paraneoplastic tissues were then downloaded and analysed through the GEO database. After screening according to fold change >2 and *p* < 0.01, we obtained 422 PLC-related targets(Figures 3A,B). We then used the BisoGenet plugin to perform PPI analysis on the above disease targets, yielding a total of 6,126 nodes and 134,394 edges. Next, to more accurately predict the potential core targets of HSG in the treatment of PLC, we used the CytoNCA plugin in Cytoscape software to systematically integrate the PPI analysis of drug targets and disease targets. After sequentially setting the conditions of DC > 56 and DC > 98; BC > 4.68 × 10^−4^; and CC > 0.447, the core mining of the integrated network was performed at the topological level. Finally, 362 potential core targets were obtained (Figure 4).

**FIGURE 5:**
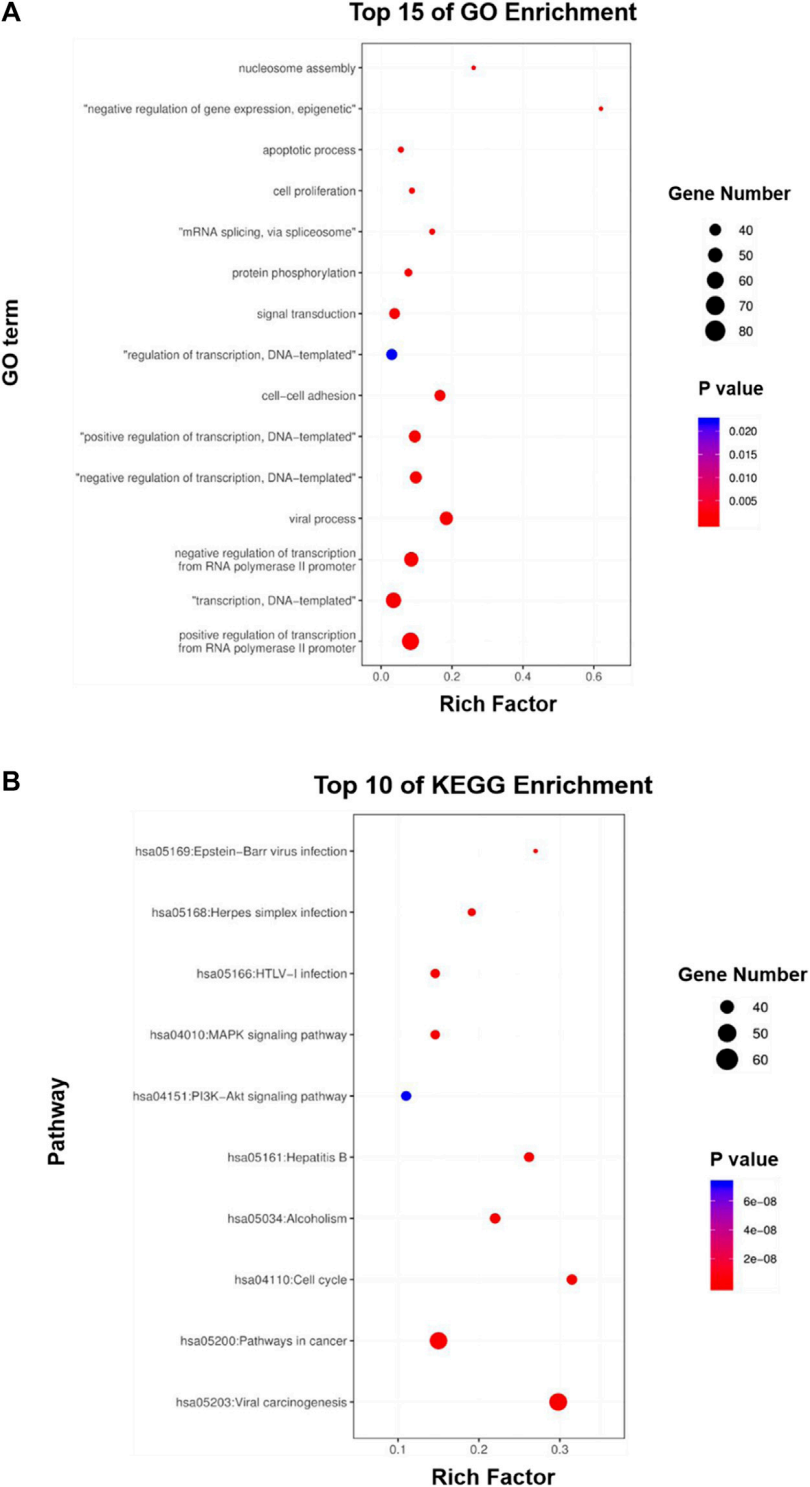
GO and KEGG enrichment analyses of the core targets for HSG against PLC. **(A, B)** The core targets that were enriched in different biological processes (GO-BP) and signaling pathways (KEGG) as assessed by DAVID v6.8 (*p*-value <0.05).

To further predict the potential biological functions and molecular mechanisms involved in the above core targets, we used DAVID v6.8 to perform enrichment analyses of the biological processes of the Gene Ontology database (GO-BP) and the molecular mechanisms of the Kyoto Encyclopedia of Genes and Genomes (KEGG) on the above 362 core targets. GO-BP analysis showed that the above core targets were mainly closely related to transcription, signal transduction, cell proliferation and apoptosis (Figure 5A), while the KEGG analysis showed that the above core targets were mainly closely related to viral carcinogenesis, pathways in cancer, cell cycle, PI3K-Akt signaling pathway and MAPK signaling pathway (Figure 5B). Therefore, the results of these *in silico* analyses provide prospective guidance for revealing and verifying the pharmacological mechanisms of HSG in the treatment of PLC.

### 3.5 Huatan Sanjie Granules could significantly reduce the growth viability and motility of LC cells

**FIGURE 6:**
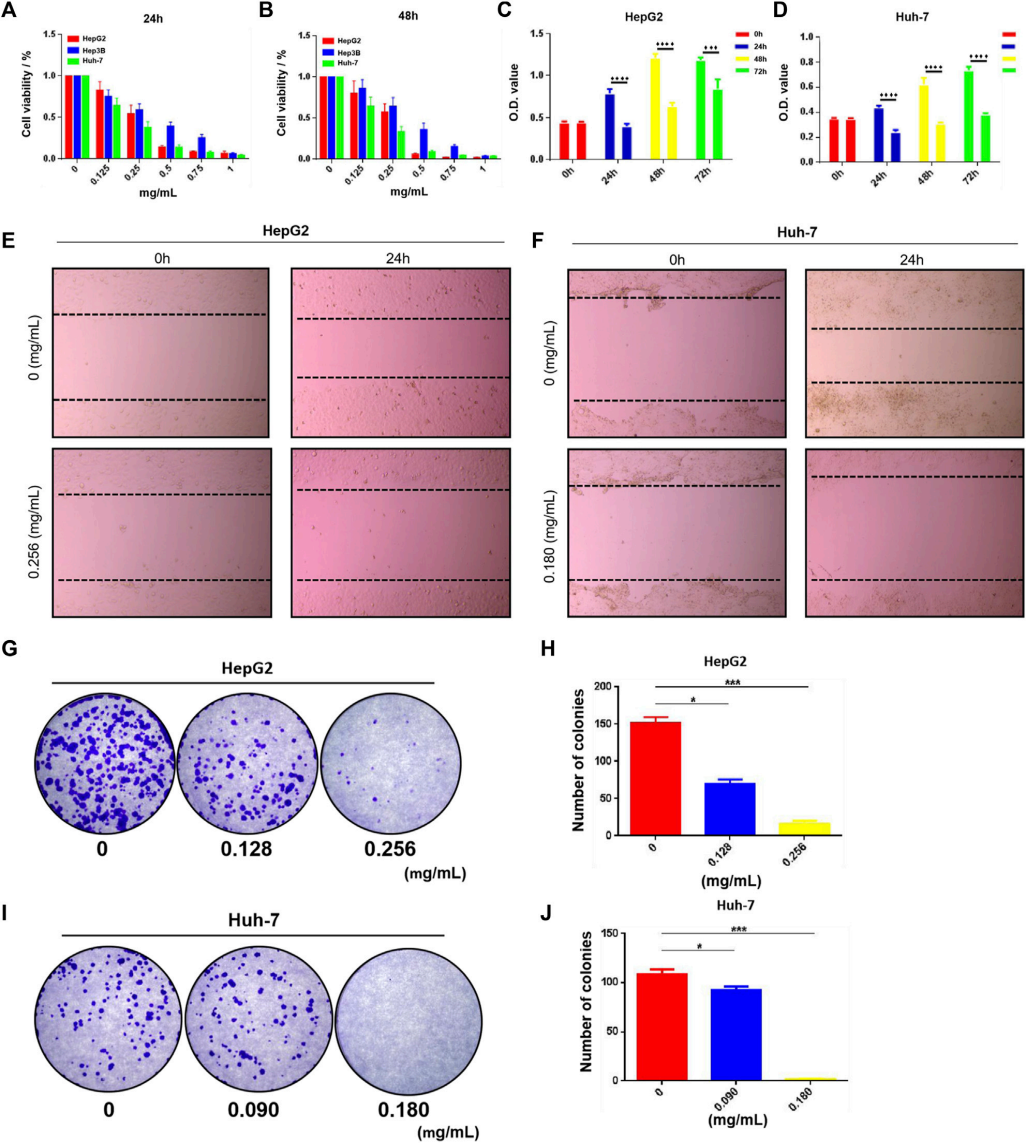
HSG inhibited PLC cells growth and suppressed its viability. **(A, B)** The CCK-8 assay was carried out to evaluate PLC cell viability upon *HSG* treatments with different dosages (0, 0.125, 0.25, 0.5, 0.75, 1 mg/mL) in 24 and 48 h. **(C, D)** CCK-8 assay was conducted to assess PLC cell growth ability after treating with *HSG* at different dosages (0.256 or 0.180 mg/mL) in 24, 48, and 72 h. **(E, F)** Wound-healing assay was used to evaluate the cell motility after *HSG* treatment (0.256 or 0.180 mg/mL) for 24 h. **(G–J)** Cell colony formation assay was carried out to access the cell clonality after HSG treatment (0.256 or 0.180 mg/mL) for 24h. *p < 0.05 and ***p < 0.001 were based on the Student's *t*-test.

We performed a series of *in vitro* pharmacodynamic evaluations on HSG using the LC cell model. First, the CCK-8 assay showed that the drug had a dose- and time-dependent inhibitory effect on the growth of HepG2, Hep3B, and Huh-7 human LC cells (Figures 6A,B). The half-maximal inhibitory concentration (IC_50_) analysis at 24 h showed that the IC_50_ values of HSG in HepG2 and Huh-7 were 0.256 ± 0.003 mg/mL and 0.181 ± 0.018 mg/mL, respectively. The cell growth viability assay showed that the viability of HepG2 and Huh-7 cells treated with the IC_50_ of HSG at 24–72 h significantly decreased compared with that of the blank control group (Figures 6C,D). The cell scratch assay showed that the motility of HepG2 and Huh-7 cells treated with IC_50_ HSG at 24 h was significantly lower than that of the blank control group (Figures 6E,F). The cell colony formation assay showed that after 24 h of treatment with the IC_50_ of HSG at 24 h, the colony forming ability of the two types of cells decreased (Figures 6G–J). The above results all indicate that HSG has a direct inhibitory effect on the survival and motility of LC cells.

### 3.6 Huatan Sanjie Granules could induce apoptosis and S phase arrest of LC cells

**FIGURE 7:**
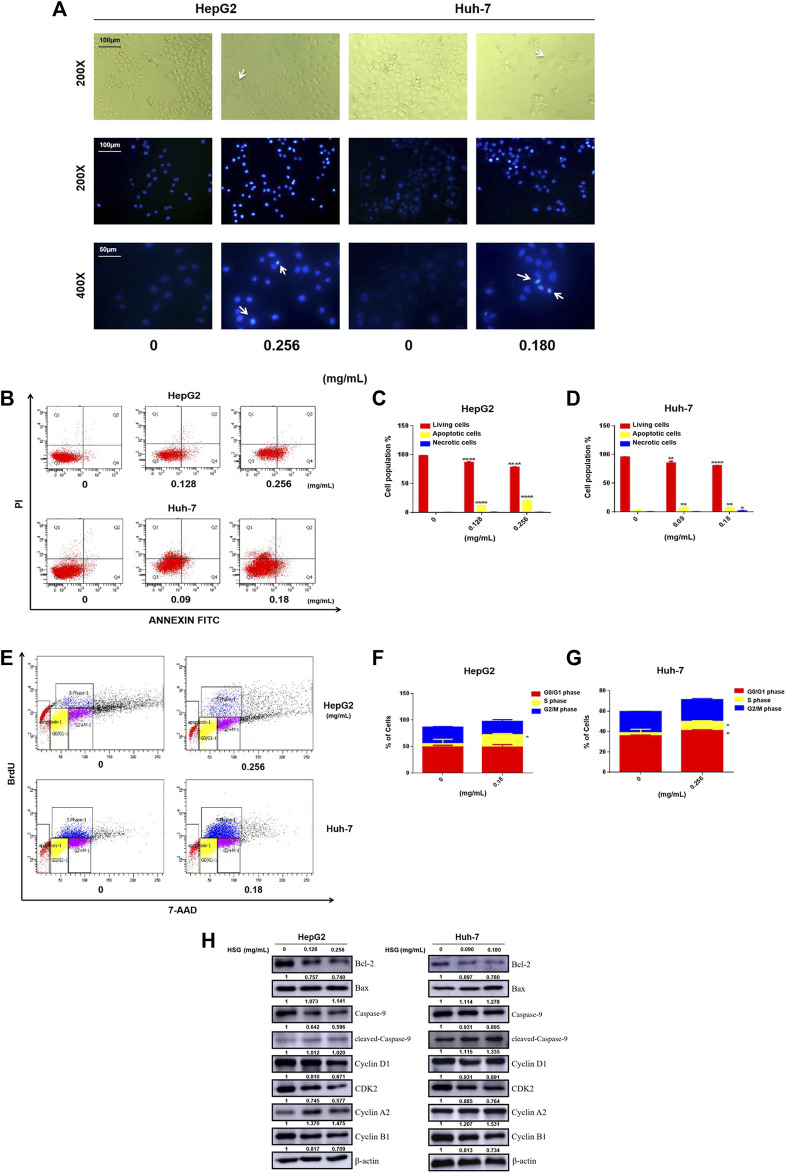
HSG treatment led to apoptosis and disturbance of cell cycle in PLC cells. **(A)** Cell morphology was observed in white light and fluorescence field. **(B)** Induced apoptosis of HepG2 and Huh-7 cells upon *HSG* treatment. The apoptotic events were evaluated by flow cytometry after above cells were treated with *HSG* at varying doses (HepG2: 0, 0.128 and 0.256 mg/mL, Huh-7: 0, 0.09 and 0.18 mg/mL) for 24 h. **(C, D)** Statistical analyses of apoptotic rates in above cells. *p < 0.05, **p < 0.01 and ****p < 0.0001 were based on the Student's *t*-test. **(E)** Cell cycle assessment after *HSG* treatment in HepG2 (0 and 0.256 mg/mL) and Huh-7 (0 and 0.18 mg/mL) for 24h by using flow cytometry. **(F, G)** Statistical analysis of the proportions of cells in different phases using above cells. *p < 0.05 was based on the Student's *t*-test. **(H)** The PLC cells were treated with *HSG* at different concentrations for 24 h. Expression levels of Bcl-2, Bax, pan-caspase-9, cleaved-caspase-9, CyclinA2, Cyclin D1, CDK2, and CyclinB1 were analyzed by Western blot.

The cell functional experiments demonstrated that the morphology of LC cells after 24 h of treatment with HSG showed significant changes. Compared with the blank control group, the two types of LC cells showed typical apoptosis characteristics. For instance, the cells became wrinkled, and their morphology became round. Furthermore, fluorescence observation after Hoechst 33,342 staining demonstrated that the nuclei after HSG treatment showed dense staining (Figure 7A). Apoptotic cell population stained with Annexin V-FITC was markedly dose-dependently elevated after HSG treatment (Figures 7B–D). Western blot (WB) analysis suggested that HSG promoted the accumulation of the pro-apoptotic protein Bax and cleaved Caspase-9, while the expression level of the anti-apoptotic protein Bcl-2 was downregulated by the HSG in a dose-dependent manner (Figure 7H; Supplementary Figure S1).

Meanwhile, cell cycle analysis with BrdU incorporation by flow cytometry showed that HSG arrested both HepG2 and Huh-7 cells in S phase, thereby significantly inhibiting their growth and proliferation activity (Figures 7E–G). Consistent with this result, WB analysis also showed accumulation of S-phase specific marker cyclin A2 and downregulation of cyclin D1, CDK2, and cyclin B1 in LC cells treated with HSG (Figure 7H; Supplementary Figure S1). Therefore, the above results all indicate that HSG could inhibit cell growth and viability by inducing apoptosis and cell cycle arrest in PLC.

### 3.7 Huatan Sanjie Granules could inhibit the growth of primary liver carcinoma cells by blocking the PI3K-Akt/MAPK signaling pathways

**FIGURE 8:**
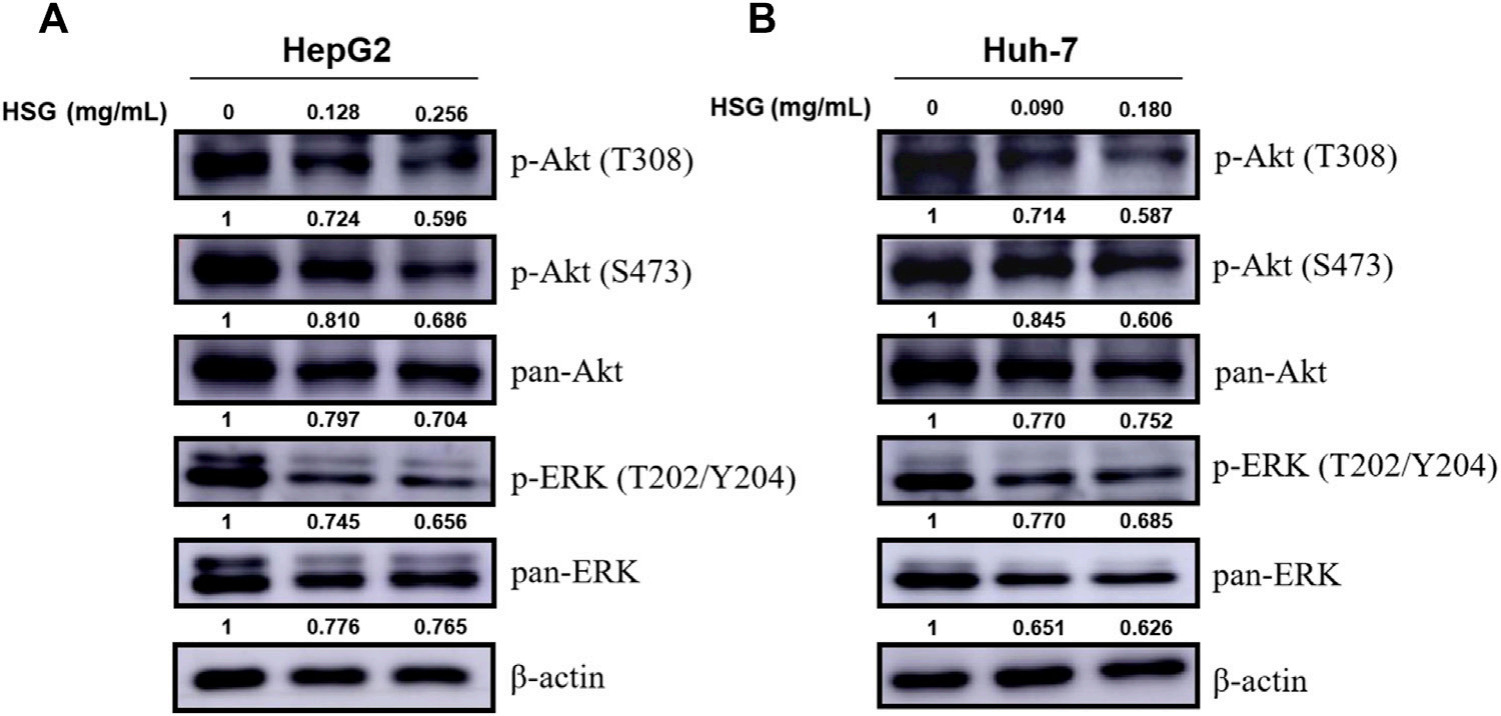
Down-regulation of p-Akt and p-ERK levels in PLC cells by HSG treatment. PLC cells were treated with HSG at varying doses for 24 h. The levels of p-Akt (T308 & S473), pan-Akt, p-ERK (T202/Y204) and pan-ERK were evaluated by western blot in **(A)** HepG2 and **(B)** Huh-7 cells, respectively.

To further explore the underlying molecular mechanisms of HSG in inhibiting the growth of PLC, we next investigated and verified the key signaling pathways involved in cell proliferation and viability. Among them, we selected the PI3K-Akt and MAPK signaling pathways based on the results of the enrichment analysis of KEGG pathways. Intriguingly, the WB results showed that HSG significantly decreased the expression levels of key factors in the above pathways after treating two PLC cells, such as p-Akt (T308 & S473) and p-ERK (T202/Y204) (Figure 8; Supplementary Figure S2). Therefore, the effect of HSG of inducing apoptosis and cell cycle arrest and inhibiting the growth of LC cells may be the result of simultaneous inhibition of the Akt and ERK signaling pathways, which indicates that HSG shares the typical TCM pharmacological characteristics of multi-ingredient, multi-target, and multi-function.

### 3.8 Huatan Sanjie Granules could inhibit important targets associated with hepatitis B virus signaling pathway

**FIGURE 9:**
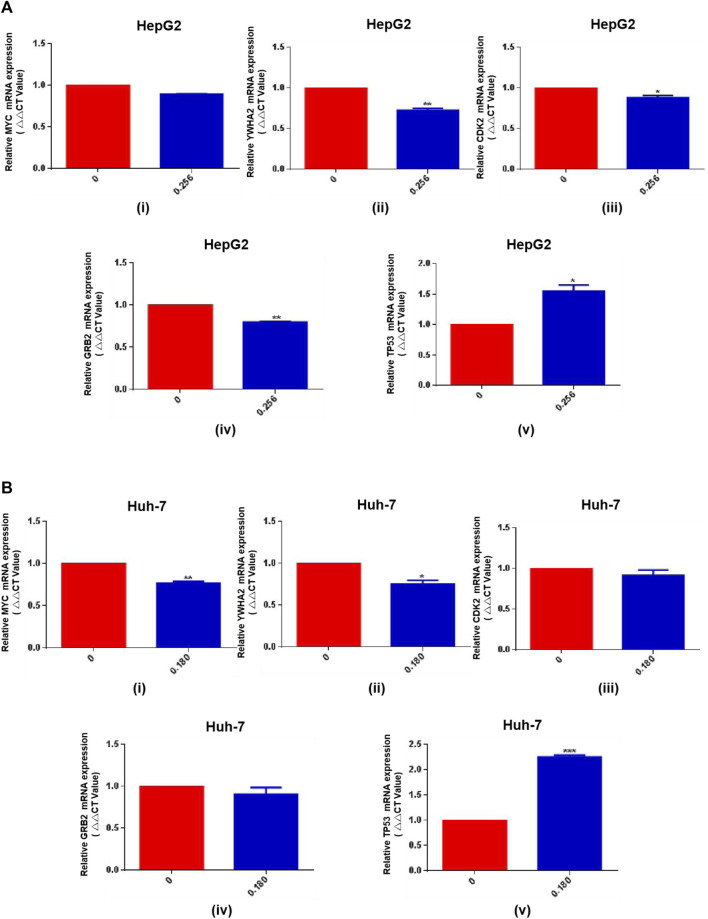
The qRT-PCR results for HSG treatment of PLC cells. **(A, B)** HSG had an improved regulatory effect on the relevant targets in HepG2 and Huh-7 cells, such as TP53 and YWHA2. *p < 0.05, **p < 0.01 and ***p < 0.001 were based on the Student's *t*-test.

Since HSG has a good efficacy against PLC in clinical practice, and most cases of PLC are closely related to the history of hepatitis B. Next, we further used HSG to intervene in and detect important HBV-related targets. qRT-PCR results showed that HSG had an improved regulatory effect on the relevant targets, such as TP53 and YWHA2. The specific results were shown in Figures 9A,B.

## 4 Discussion

In recent years, Several retrospective studies have shown that combined treatment with traditional Chinese medicine can improve the survival rate of patients with unresectable liver cancer (Tang et al., 2016; Tang et al., 2018); However, these studies mostly belong to supporting early and mid-stage liver cancer, and most TCM treatment principles are based on invigorating spleen and qi, promoting blood circulation and removing blood stasis, clearing heat and detoxifying. For example, Shenqi Fuzheng injection can enhance the inhibitory effect of (interferon-α) IFN-α on HCC cells by decreasing the expression of vascular endothelial growth factor (VEGF) (Choa et al., 2019); Alternatively, FuzhengXiaoji prescription can be realized by improving inflammatory state and blocking Ras/MAPK and Ras/PI3K-Akt signaling pathways (Liu et al., 2022) and Jiedu granules can inhibit the migration and invasion of HCC cells by regulating Smad23-dependent and independent pathways (Liang et al., 2018). Another, our HSG is based on the TCM theory of “sputum poisoning” in PLC. It is composed of SCG, YJ, JH, DNX, ZBM, and TBM. Starting from clearing heat, moving *qi*, drying dampness, and resolving phlegm, HSG has unique efficacy in the comprehensive treatment of PLC in clinical practice which the results showed the progress in terms of survival benefit, especially in the patients were BCLC stage C. Because the CHMs are a complex combination of chemical systems and disease-syndrome systems, so we are using the concepts and methods of network targeting in network pharmacology, we construct a PPI systematic network of HSG against PLC consisting of 362 putative core therapeutic targets. Meanwhile, enrichment analyses showed that HSG might induce apoptosis and cell cycle arrest of LC cells by blocking the PI3K-Akt/MAPK molecular signaling pathways. Through this study, we will further explore the adjuvant treatment effect of TCM on LC, and provide a more reliable basis for the combined treatment of TCM and Western medicine treatment.

Tumors are closely associated with excessive cell proliferation and inhibition of cell apoptosis. Therefore, cell viability assays are often used to evaluate the efficacy of anticancer drugs. In our study, HSG showed a dose- and time-dependent inhibitory effect on the growth of HepG2, Hep3B, and Huh-7 cells. Among them, the growth and motility of HepG2 and Huh-7 cells were significantly reduced compared to those of the blank control group. The colony formation ability of these cells decreased in a dose-dependent manner. These results all indicate that HSG has a direct inhibitory effect on the survival and motility of LC cells *in vitro*.

Apoptosis is an important biological process to tumor growth, as induction of apoptosis can inhibit the progression of cancer. Apoptosis is regulated by the Bcl-2 family and the Caspase family proteins (Brentnall et al., 2013). Bax is a pro-apoptotic protein in the Bcl-2 family (Chiang et al., 2011). Bcl-2 family members are key regulatory factors for cell apoptosis. Bcl-2 promotes cell survival, and its overexpression is associated with cancer. The search for molecular substances that directly target members of the Bcl-2 family, including the Bcl-2 gene itself, is leading to new cancer treatments (Kelly and strasser, 2011). Caspase-9, a protein in the caspase family, induces apoptosis through the regulation of mitochondrial morphological changes and reactive oxygen species (ROS) production (Brentnall et al., 2013). Our cytometry assay showed that apoptotic cell population was markedly dose-dependently elevated after HSG treatment. WB results also showed that HSG promoted the accumulation of pro-apoptotic protein Bax and cleavage of caspase-9, while the expression level of the anti-apoptotic protein Bcl-2 was downregulated by the drug in a dose-dependent manner. Cell cycle analysis showed that HSG arrested both HepG2 and Huh-7 cells in S phase, thereby significantly inhibiting their growth and proliferation activity. Consistent with this result, WB analysis also showed that the S phase-specific marker cyclin A2 accumulated and cyclin D1, CDK2, and cyclin B1 were downregulated in LC cells treated with HSG. The above results all indicate that HSG can inhibit cell growth by inducing apoptosis and cell cycle arrest in LC cells.

In this study, using a combination of TCM network pharmacology and bioinformatics, we found that the potential core targets of HSG against PLC were mainly related to viral carcinogenesis, pathways in cancer, cell cycle, PI3K-Akt and MAPK signaling pathways. The PI3K/Akt pathway is activated by phosphorylation and induces the proliferation and survival of cancer cells. Meanwhile, in mammalian cells, the ERK-related intracellular signal transduction pathway is considered the classical MAPK signal transduction pathway, and the ERK pathway also responds to a variety of growth factors through phosphorylation activation (Gholinejad et al., 2017). ERK phosphorylation can activate tumour-promoting transcription factors such as NF-κB, MYC, and β-catenin, leading to gene expression (Panahi et al., 2018; Muranen et al., 2016; Cao et al., 2019). Our WB results showed that HSG significantly reduced the expression levels of key factors, such as p-Akt and p-ERK, in the above pathways after treating either type of LC cells. Therefore, the effect of HSG of inducing apoptosis and cell cycle arrest and inhibiting the growth viability of LC cells may be the result of simultaneous inhibition of the Akt/ERK signaling pathways.

*TP53* mutation is the most common mutation in PLC and affects its progression and prognosis. This gene plays an important role in the maintenance of genomic stability, and its loss of function can lead to centrosome amplification, aneuploidy cell proliferation, and chromosome instability (Rao et al., 2017). *TP53* mutation leads to the downregulation of the immune response in PLC, which has important effects on the immune microenvironment of PLC (Long et al., 2019). Most PLC cases are associated with chronic infection with HBV and/or hepatitis C virus. Chronic HBV infection leads to long-term and low-level destruction and regeneration of hepatocytes, leading to fibrosis, cirrhosis, and steatosis and eventually to PLC (Matsuda et al., 2013). *TP53* mutations often occur in HBV-related PLC. *TP53* and *CTNNB1* mutation are causative in 25%–30% of PLC (Schulze et al., 2015). In addition, several studies have shown that *TP53* mutation is associated with a poor prognosis of PLC patients with HBV infection in China (Woo G et al., 2011). Therefore, HBV infection affects the expression of IncRNA, which also affects the stability of p53 protein (Liu et al., 2019). In addition, the functional roles of YWHA in a variety of cellular processes include signal transduction, transport, apoptosis, stress response, and malignant transformation (Dougherty and Morrison, 2004; Mackintosh, 2004; Bridges and Moorhead, 2005). In this study, qRT-PCR results showed that HSG had a positive-feedback regulatory effect on *TP53* and *YWHA2* targets, confirming that the anticancer mechanism of HSG is achieved through regulating the expression of *TP53* and explaining why HSG is more effective on PLC patients with HBV infection.

In summary, our study confirms that HSG has significant efficacy in inhibiting LC cells, through an analysis of the clinical effects of HSG as the classic TCM prescription for the treatment of PLC, an analysis of its drug composition and their functions, and biological experimental verifications. Our combination of network pharmacology and bioinformatics has provided preliminary theoretical guidance on the antitumor mechanisms of CHM prescriptions and lays a foundation for the in-depth study of those mechanisms-of-action. However, we did not further explore the key active therapeutic ingredient from HSG and related direct action targets through the CHM theory of “monarch-minister-assistant-guide.” Therefore, in future research, we plan to explore the specific active ingredients involved in the monarch drug in HSG using UPLC-MS/MS technology, and validate the relevant protein targets through target binding validation techniques (such as SPR, ITC, etc.). We believe that with the advancement of medical technology, more new ingredients and targets of classic CHMs will be discovered for the treatment of PLC, which will complement the research results of this study.

## Data Availability

The original contributions presented in the study are included in the article/Supplementary Material, further inquiries can be directed to the corresponding authors.

## Supplementary material

SUPPLEMENTARY FIGURE S1 The statistical results related to WB experiments (Bcl-2, Bax, pan-caspase-9, cleaved-caspase-9, CyclinA2, Cyclin D1, CDK2, and CyclinB). **P* < 0.05, ***P* < 0.01, ****P* < 0.001 and *****P* < 0.0001 were based on the Student’s *t*-test.

SUPPLEMENTARY FIGURE S2 The statistical results related to WB experiments (p-Akt (T308 & S473), pan-Akt, p-ERK (T202/Y204) and pan-ERK). ***P* < 0.01, ****P* < 0.001 and *****P* < 0.0001 were based on the Student’s *t*-test.

## Abbreviations

BC, betweenness centrality; BCLC, Barcelona Clinic Liver Cancer; CC, closeness centrality; CCK-8, Cell Counting Kit-8; CHM, Chinese herbal medicine; DAVID: Database for Annotation, Visualization and Integrated Discovery; DC, degree centrality; ECOG, Eastern Cooperative Oncology Group; FC, fold change; GEO, Gene Expression Omnibus; GO-BP, Gene Ontology database; HSG, Huatan Sanjie Granules; HBV, hepatitis B virus; KEGG, Kyoto Encyclopedia of Genes and Genomes; LC, liver cancer; PLC, primary liver carcinoma; PPI, protein–protein interaction; PVDF, polyvinylidine fluoride; qRT-PCR, quantitative reverse transcription-PCR; ROS, reactive oxygen species; TCM, traditional Chinese medicine; 1WB, Western blot.
