# Supplementary figures and images for: Adjuvant therapy with Huatan Sanjie Granules improves the prognosis of patients with primary liver cancer: a cohort study and the investigation of its mechanism of action based on network pharmacology
Source: Front Pharmacol. 2023 May 30;14:1091177. doi: 10.3389/fphar.2023.1091177 (PMC10267985; doi:10.3389/fphar.2023.1091177)

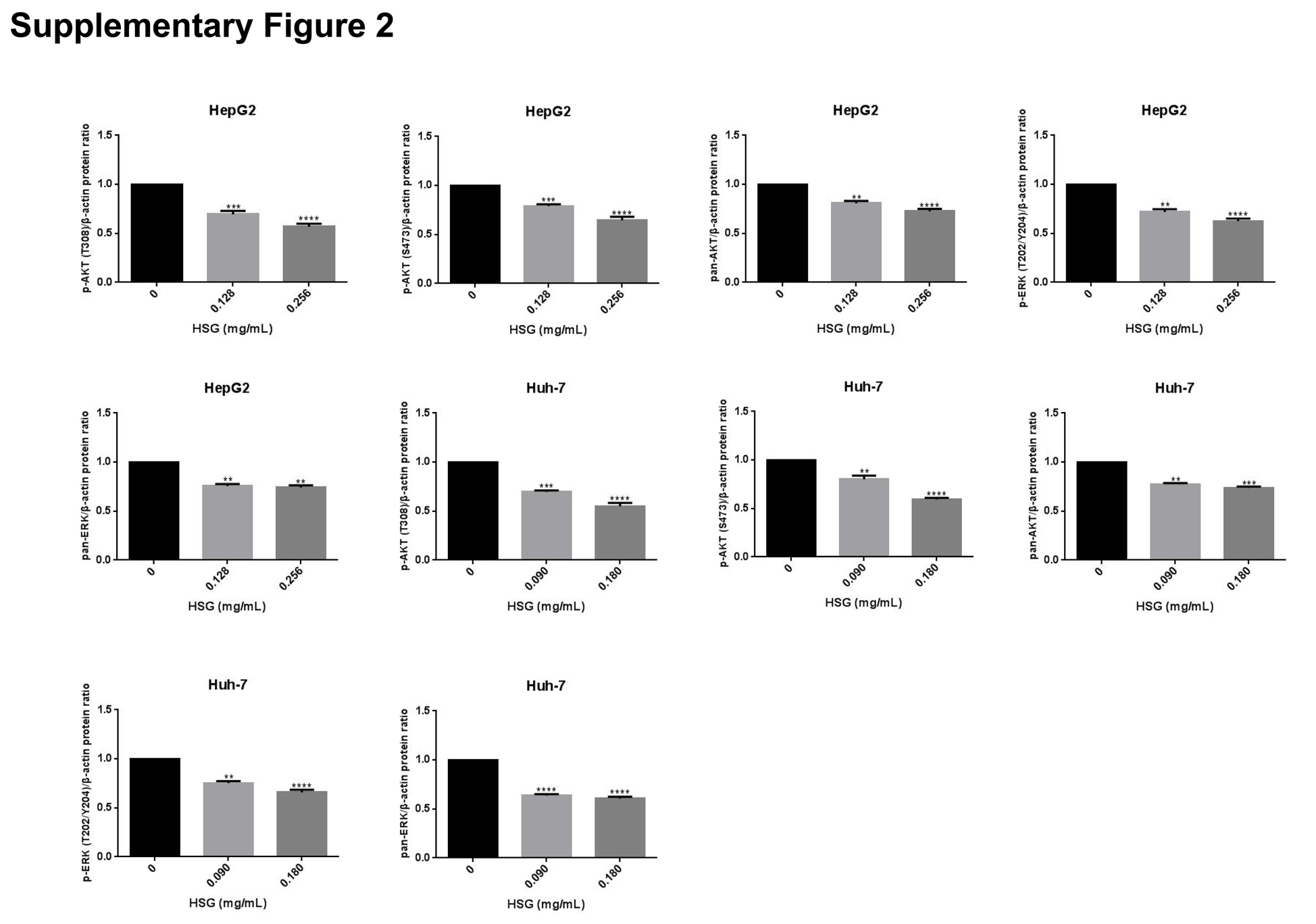

Supplement: Supplementary file 1 [file Image2.TIF]

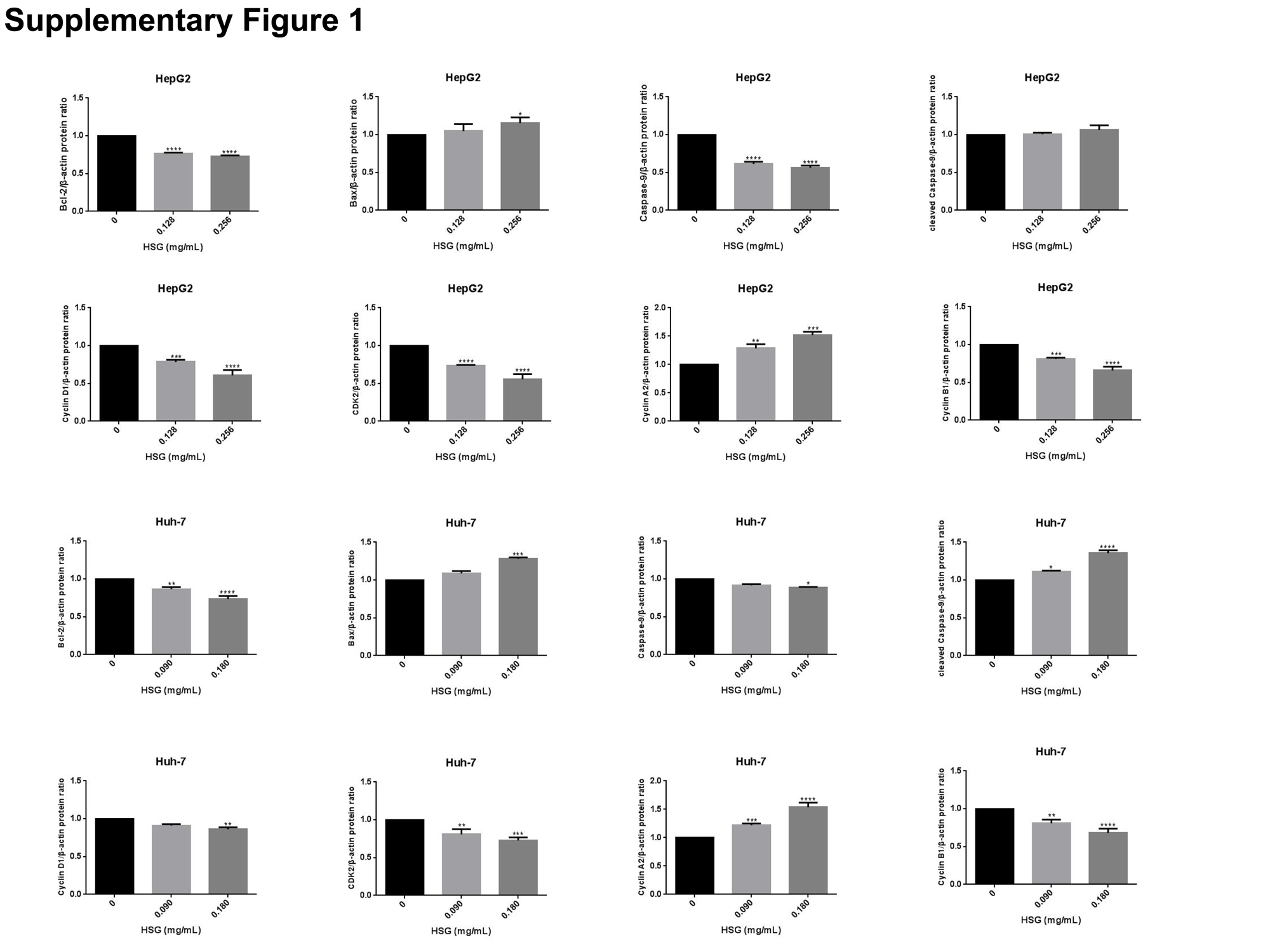

Supplement: Supplementary file 2 [file Image1.TIF]
